# Recurrent candidiasis and early-onset gastric cancer in a patient with a genetically defined partial MYD88 defect

**DOI:** 10.1007/s10689-015-9859-z

**Published:** 2015-12-23

**Authors:** Ingrid P. Vogelaar, Marjolijn J. L. Ligtenberg, Rachel S. van der Post, Richarda M. de Voer, C. Marleen Kets, Trees J. G. Jansen, Liesbeth Jacobs, Gerty Schreibelt, International Gastric Cancer Genetics Group, I. Jolanda M. de Vries, Mihai G. Netea, Nicoline Hoogerbrugge

**Affiliations:** Department of Human Genetics, Radboud university medical center, P.O. Box 9101, 6500 HB Nijmegen, The Netherlands; Department of Pathology, Radboud university medical center, Nijmegen, The Netherlands; Department of Internal Medicine, Radboud university medical center, Nijmegen, The Netherlands; Department of Tumor Immunology, Radboud university medical center, Nijmegen, The Netherlands; Department of Medical Oncology, Radboud university medical center, Nijmegen, The Netherlands

**Keywords:** *MYD88*, Gastric cancer, *Candidaalbicans*, Interleukin-17, Th17 response

## Abstract

Gastric cancer is caused by both genetic and environmental factors. A woman who suffered from recurrent candidiasis throughout her life developed diffuse-type gastric cancer at the age of 23 years. Using whole-exome sequencing we identified a germline homozygous missense variant in *MYD88*. Immunological assays on peripheral blood mononuclear cells revealed an impaired immune response upon stimulation with *Candida albicans*, characterized by a defective production of the cytokine interleukin-17. Our data suggest that a genetic defect in *MYD88* results in an impaired immune response and may increase gastric cancer risk.

## Introduction

GC is a multifactorial disease with a low survival rate in which both genetic and environmental factors are involved. Infection with *Helicobacter pylori* is an important risk factor for GC [1].

Approximately 1–3 % of GC cases fulfill the criteria for Hereditary Diffuse Gastric Cancer (HDGC; MIM 137215), a cancer predisposition syndrome characterized by early-onset diffuse GC. In approximately 20 % of the families fulfilling the HDGC criteria a germline mutation can be found in the *CDH1* gene encoding the E-cadherin protein [2–4]. Recently also *CTNNA1* and *MAP3K6* are described as putative gastric cancer predisposition genes [5, 6], but in the majority of cases the underlying genetic cause remains unknown. In this study we report the case of a 23-year-old woman with diffuse-type GC and recurrent candidiasis, in whom we identified a novel germline homozygous *MYD88* variant.

## Materials and methods

### Genetic analysis of the 23-year-old gastric cancer patient

Genomic DNA was extracted from peripheral blood cells and hybridized to Affymetrix SNP6.0 arrays (Affymetrix, Santa Clara, CA, USA) according to the manufacturer’s protocol. The genotype was generated using the Birdseed analysis software incorporated in the Affymetrix Genotyping Console v2.1 (Affymetrix). Detected copy number variants (CNVs) were compared to a set of healthy controls, as described previously [7], to exclude regions of normal variation.

Massive parallel whole-exome sequencing was performed after DNA enrichment using the human SureSelect 50 Mb kit (version 2, Agilent Technologies, Santa Clara, CA, United States) on a 5500XL SOLiD platform (Life Technologies, Bleiswijk, The Netherlands). Reads were mapped to the hg19 reference genome using SOLiD BioScope software (Life Technologies) [8]. Variants were annotated using an in-house annotation pipeline, as described previously [8]. Briefly, from our total set of variants we selected high-confidence (≥5 variant reads and/or ≥20 % variant reads) non-synonymous variants with a SNP frequency in dbSNP (v132) below 1 % that were present at most once in our in-house variant database containing 2096 exomes (the majority of European ancestry) [8].

Missense variants were considered putatively pathogenic when the affected nucleotide was highly conserved (PhyloP ≥ 3.0) and if two of the following three in silico prediction programs considered this variant deleterious/damaging: SIFT [9], PolyPhen-2 [10], and Align GVGD [11] [all incorporated in the Alamut 2.0 software package (Interactive Biosoftware, Rouen, France)].

### Cohort screening for MYD88 variants

Our cohort of gastric cancer patients consisted of 126 additional *CDH1* mutation-negative index patients that meet one of the 2010 HDGC criteria, with the exception that all types of GC histology were included [12]. Germline DNA of these patients was analyzed for mutations in *MYD88* (NM_001172567.1) using either Sanger sequencing (n = 41), ion semiconductor sequencing (n = 63) (PGM, Life Technologies) or whole-exome sequencing (n = 22, see above). A cohort of 183 healthy individuals from Turkish or Pakistani descent were analyzed for the *MYD88* variant using Sanger sequencing.

For Sanger sequencing the full coding sequence of *MYD88*, including splice junctions, was amplified using polymerase chain reaction (primer sequences and PCR conditions are available on request) and screened for mutations using Big-Dye terminator sequencing (BigDye Terminators (v 1.1) Applied Biosystems, USA). Analysis was performed on an ABI 3730 DNA Analyzer (Applied Biosystems). Subsequently, data was analyzed for variants using Chromas Lite (Technelysium, South Brisbane, Australia).

Ion semiconductor sequencing was performed using a custom designed multiplex Ion AmpliSeq™ PCR primer panel (Life Technologies) according to the manufacturer’s protocol. Briefly, for the library preparation, four DNA samples from our gastric cancer patient cohort were pooled equimolarly and 10 ng of DNA was used for AmpliSeq amplification. Each pool was barcoded with Ion Xpress Barcode adapters (Life Technologies). The barcoded libraries were purified using Agencourt AMPure XP beads (Beckman Coulter Genomics, High Wycombe, UK), pooled and diluted for use in a 200-bp amplification run on an OneTouch emulsion PCR system (Life Technologies). Before loading onto the 316 chip, sequencing primer and polymerase were added to the final enriched spheres. SeqNext software (JSI Medical Systems) was used for mapping and analysis of the data. All amplicons were analyzed at a coverage of at least 150×. The identified variant was validated in the individual DNA samples of the pool using Sanger sequencing as described above.

### Immunological assays

The cytokine production capacity was assessed as previously described [13]. Briefly, venous blood was collected into EDTA tubes and primary blood mononuclear cells (PBMCs) were isolated by density centrifugation of blood diluted 1:1 in PBS over Ficoll-Paque (Pharmacia Biotech AB, Uppsala, Sweden). Cells were washed three times with PBS and resuspended in RPMI 1640 (Dutch modified) supplemented with 50 mg/L gentamicin, 2 mM l-glutamine, and 1 mM pyruvate. Cells were counted on a Coulter Counter Z (Beckman Coulter, Mijdrecht, the Netherlands) and adjusted to 5 × 10^6^ cells/mL. PBMCs (5 × 10^5^) in a 100 μL volume were added to round-bottom 96-well plates (Greiner, Alphen a/d Rijn, the Netherlands) and incubated with either 100 μL of culture medium (negative control) or one of the stimuli described for 24 h (TNFα, IL-6), 48 h (IFN-γ) and/or 7 days (IL-17 and IL-22). Cytokine production was tested after stimulation of PBMCs with: lipopolysaccharide (LPS) derived from *E. coli* (10 ng/mL), heat-killed *C. albicans* (10^5^ microorganisms/mL), heat-killed *H. pylori* (10^7^ microorganisms/mL), heat-killed *S. aureus* (10^7^ microorganisms/mL), recombinant IL-1β or IL-23 (100 ng/mL), and recombinant IL-12 or IL-18 (10 ng/mL) after 24 h and 7 days. 10 % human pooled serum was added when PBMCs were incubated during 7 days. After incubation the supernatants were stored at −20 °C until assay. Experiments were performed in duplicate.

## Results

### Early-onset gastric cancer patient with extensive infectious phenotype

**Fig. 1:**
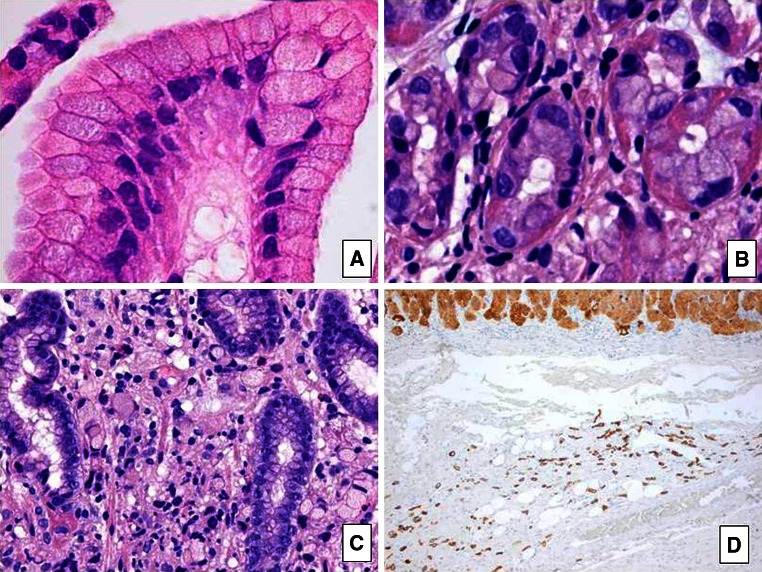
Signet ring cell carcinoma of the patient. *Note* the typical spreading of single cells in the gastric glands (**a**, **b**) and invasive signet ring cells between glands in the lamina propria (**c**). Gastrectomy shows patchy remnants of cancer cells in the submucosa (**d** cytokeratin 8.18 stain) [original magnifications 400× (**a**–**c**); 50× (**d**)]

The patient first presented with pain in the left upper abdomen at the age of 23 years. She was diagnosed with an *H. pylori* infection, for which eradication treatment was given. As the abdominal pain continued, a gastroduodenoscopy was performed, which revealed a gastric ulcer. Histopathological analyses of biopsies showed a signet ring cell carcinoma and moderate chronic active, non-specific gastritis (Fig. 1a–c). The patient was treated with neoadjuvant chemotherapy resulting in almost complete regression of the gastric tumor at the time of surgery (Fig. 1d).

Because of the early-onset diffuse GC the patient was referred for genetic counseling. Family history revealed distantly related parents of Kurdish descent, six unaffected siblings and no other relatives with GC. Her medical history revealed that she lost her fingernails (onycholysis) at the age of two years. Throughout her life, she suffered from recurrent vaginal *Candidaalbicans* infections and recurrent fungal infections, in particular dermatophytic onychomycosis, of which at clinical examination pigmented rash could be observed in her neck, between her breasts and at the rim of her fingernails. Her medical history was negative for allergies and pyogenic infections.

### Identification of a novel homozygous missense variant in MYD88

The patient developed diffuse-type GC at a very young age and therefore meets the *CDH1* testing criteria [12]. *CDH1* germline mutation analysis was performed, but no germline *CDH1* alterations were found by sequencing analysis of the entire open reading frame, including intron–exon boundaries, or Multiplex Ligation-dependent Probe Amplification (MLPA) of all exons. Therefore, further research on possible underlying genetic aberrations was performed. Copy number profiling using SNP6.0 arrays showed no significant copy number variations, but revealed large areas with loss of heterozygous calls, in agreement with parental consanguinity (data not shown).

Subsequently, whole-exome sequencing on germline DNA from the patient was performed. No putative pathogenic mutations in other cancer predisposition genes among which *CTNNA1* and *MAP3K6* were detected. The heterozygous missense variant in *PMS1* that was validated using Sanger sequencing (Table 1) probably does not contribute to the phenotype of the patient. Because the parents were distantly related, we focused our data-analysis on homozygous variants with a variant percentage ≥80 % (n = 17). Five variants that were putatively pathogenic (one protein-truncating variant and four missense variants) were validated using Sanger sequencing, of which the missense variant (c.712C>T, p.(Arg238Cys)) in the myeloid differentiation primary response 88 (*MYD88*) gene was considered most relevant. This variant affects a moderately conserved nucleotide (phyloP 3.76) of a highly conserved amino acid in the Toll/interleukin-1 receptor (TIR) homology domain of the gene and is predicted to be damaging to protein function using in silico prediction programs. For the list of all validated variants and occurrence of variation in the corresponding genes in control databases, see Table 1.

**Table 1:** Variants identified by exome sequencing and validated using Sanger sequencing

Gene	Variant	% variation	Number of different: truncating variants second in-house dataset^a^/homozygous missense variants second in-house dataset^a^/truncating variants EVS^b^	Variant in EVS	In silico analysis	Gene function^c^
*Variants in an in*-*house generated list of hereditary cancer predisposing genes*						
PMS1 (NM_000534)	chr2:190742047 A>G (p.(E895G))	63.93443 (39/61 reads)	1/1/4	No	PhyloP: 3.72 Grantham Score: 98 Align GVGD: C65 SIFT: Deleterious PolyPhen: Probably damaging	Belongs to the DNA mismatch repair mutL/hexB family. PMS1 is thought to be involved in the repair of DNA mismatches. It can form heterodimers with MLH1, a known DNA mismatch repair protein Mutations in this gene cause hereditary nonpolyposis colorectal cancer type 3 (HNPCC3) (either alone or in combination with mutations in other genes involved in the HNPCC phenotype)
*Homozygous variants in the patient identified by whole*-*exome sequencing*						
COL7A1 (NM_000094)	chr3:48602231 G>A (p.(Q2935*))^d^	100 (8/8 reads)	3/4/7	No	PhyloP 1.665 Grantham Score: 1000 Align GVGD: NA SIFT: NA PolyPhen: NA	Collagen, type VII, alpha 1. Functions as an anchoring fibril between the external epithelia and the underlying stroma
MYD88 (NM_001172567)	chr3:38182252 C>T (p.(R230C))	100 (81/81 reads)	0/0/0	No	PhyloP: 3.838 Grantham Score: 180 Align GVGD: C15 SIFT: Deleterious PolyPhen: Probably damaging	Myeloid differentiation primary response 88. Plays a central role in the innate and adaptive immune response. Functions as a signal transducer in the interleukin-1 and Toll-like receptor signaling pathways. Patients with defects in MYD88 have an increased susceptibility to pyogenic bacterial infections
EVX2 (NM_001080458)	chr2:176948437 T>G (p.(K23T))	97.14286 (34/35 reads)	0/2/0	No	PhyloP: 4.521 Grantham Score: 78 Align GVGD: C0 SIFT: Deleterious PolyPhen: Probably damaging	Even-skipped homeobox 2. Homeobox transcription factor that is related to the protein encoded by the Drosophila even-skipped (eve) gene, a member of the pair-rule class of segmentation genes.
ITIH1 (NM_002215)	chr3:52818372 C>T (p.(P141L))	100 (24/24 reads)	4/0/5	No	PhyloP: 5.467 Grantham Score: 98 Align GVGD: C0 SIFT: Deleterious PolyPhen: Probably damaging	Inter-alpha-trypsin inhibitor heavy chain 1. The protein encoded by this gene is the heavy chain of a serine protease inhibitor that may serve to carry hyaluronan in plasma
PRKAR2A (NM_004157)	chr3:48789705 G>A (p.(R329C))	99.22481 (128/129 reads)	0/0/0	Yes, 3 times heterozygous in EA population	PhyloP: 5.974 Grantham Score: 180 Align GVGD: C35 SIFT: Deleterious PolyPhen: Probably damaging	Protein kinase, cAMP-dependent, regulatory, type II, alpha. cAMP is a signaling molecule important for a variety of cellular functions. This subunit has been shown to regulate protein transport from endosomes to the Golgi apparatus and further to the endoplasmic reticulum (ER)

*STOP* variant resulting in a premature termination codon, *NA* not applicable, *NSMIS* nonsynonymous missense variant, *EA* European American population

^a^This database differs from the database that was used to filter the exome data and contains high-coverage paired-end exome sequencing data of 2329 individuals that are not suspected to have a form of hereditary cancer

^b^See Ref. [14]

^c^See Ref. [26]

^d^The variant (p.(Q2935*)) in *COL7A1* is located in the last 50 base pairs of the second to last exon of the gene. Therefore, nonsense mediated decay is not expected here

### Variation in the MYD88 gene in healthy controls and an additional cohort of gastric cancer patients is rare

To assess the variation in *MYD88* in control databases, we extracted all the germline variation in the coding region from *MYD88* from a second in-house dataset and the Exome Variant Server (EVS) [14]. Furthermore, we used The Cancer Genome Atlas (containing 220 gastric adenocarcinomas) to determine the amount of somatic variation in the *MYD88* gene [15]. Germline and somatic variation in the coding region of *MYD88* is rare with less than 0.5 % heterozygous missense mutations in about 8832 individuals. The variant detected in our patient is not present in any of the commonly used control databases nor in 183 healthy controls from Turkish or Pakistani descent. Moreover, only one other heterozygous *MYD88* variant (c.704G>A, p.(G235D)) was identified by targeted resequencing of the coding regions of the gene in 126 additional patients with early-onset and/or familial GC. In silico analyses predicted this variant to be benign. These results indicate that variants in *MYD88* are infrequent in both controls and GC patients.

### Immunological assays reveal functional relevance of the MYD88 variant

**Fig. 2:**
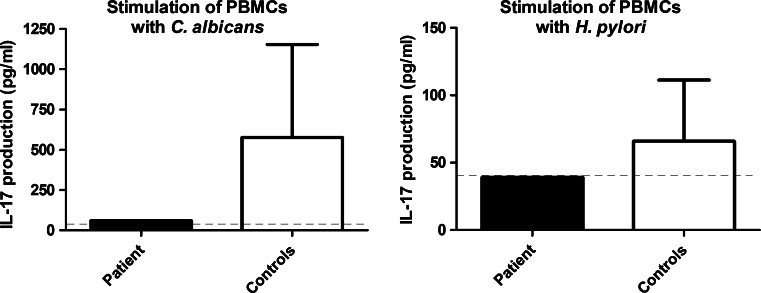
Immunological assays on peripheral blood mononuclear cells (PBMCs) from the patient with the *MYD88* variant. Assays revealed an impaired immune response upon stimulation with *C. albicans* (*left panel*), characterized by a specific defect in production of the Th17 cytokine IL-17. Immune response to *Helicobacter pylori* was normal (*right panel*). The detection limit for the assay is indicated with the *dotted line*. Values depicted are the means with standard deviation (SD)

Since MYD88 plays a central role in the immune response, we analyzed the cytokine production of peripheral blood mononuclear cells (PBMCs) of the patient, stimulated with heat-killed *C. albicans* and *H. pylori*. As expected based on the infections seen in patients with MYD88 deficiency [16], a specific defect in the production of Th17 cytokine IL-17 (Fig. 2, left panel) was observed upon re-stimulation of the patients’ PBMCs with *C. albicans*. In healthy individuals that have been exposed to *H.pylori*, an enhanced IL-17 production is observed after re-stimulation of the PBMCs with this pathogen [17]. When we performed this assay for the patient, no difference was observed compared to controls (Fig. 2, right panel). The immune response to Toll-like receptor stimuli and *S. aureus* was normal (data not shown).

## Discussion

In this study we report the case of a young woman with diffuse-type GC in which we did not find a germline mutation in the known cancer predisposition genes like *CDH1*, *CTTNA1* and *MAP3K6* that are associated with diffuse gastric cancer susceptibility. Using whole-exome sequencing, we identified a novel germline homozygous *MYD88* variant, which probably explains the patient’s vulnerability to *C. albicans* and other infections that might have contributed to the development of her gastric cancer.

MYD88 plays a central role as a mediator of the innate immune response to infections. The infectious phenotype of autosomal recessive MYD88 deficiency (OMIM 612260) is dominated by invasive pyogenic infections and the main bacteria isolated in cases of invasive infection are *Staphylococcus pneumoniae*, *S. aureus*, and *Pseudomonas aeruginosa.* Also fungal infections, for example caused by the yeast *C. albicans*, are described in patients with MYD88 deficiency [18]. In mice it was shown that MYD88 deficiency leads to an early and rapid development of *H. pylori*-induced gastric dysplasia [19].

GC was not reported in the twenty-four patients described with MYD88 deficiency, of which the oldest patient was aged 20 at time of publication [16]. However, ‘classic’ MYD88 deficiency has a high mortality at an early age, and therefore patients likely die too young to develop GC [20]. In contrast to the functional analyses described for these MYD88 deficiency patients, our assays indicate that our patient suffers from a milder form of MYD88 deficiency, which matches her clinical phenotype and is probably explained by the type of *MYD88* mutation, which to the best of our knowledge was not described before.

Therefore, we hypothesize that, in our patient, the partial MYD88 defect causes an impaired immune response, resulting in recurrent *C. albicans* infections and nonspecific gastritis increasing the risk of signet ring cell carcinoma of the stomach wall. Whether GC is more frequently diagnosed in patients with recurrent fungal infections is unknown, but it is known that patients with simultaneous *C. albicans* and *H. pylori* infection have an increased risk of developing gastric ulcers [21]. *C. albicans* is able to degrade E-cadherin in vitro [22], which may be a tumor initiating event in the development of gastric signet ring cell carcinoma similar to E-cadherin inactivation by mutations in its gene *CDH1*. In line with our hypothesis that impaired immune response might lead to an increased risk of gastric cancer, the co-occurrence of *C. albicans* and signet ring cell carcinoma of the stomach was previously described in a patient with acquired immune deficiency syndrome [23], who as a group have a 1.5-fold increased risk to develop GC [24]. Moreover, a tenfold increased GC risk is seen in patients with common variable immunodeficiency disorders (CVIDs) [25].

Due to neoadjuvant chemotherapy the patient received prior to surgery, no molecular analyses were possible on resection material. Therefore, we cannot exclude that, although this has not been described for other DGC patients thus far, two somatic mutations in either *CDH1* or one of the recently described candidate genes *CTNNA1* or *MAP3K6* [5, 6] are causal for the DGC development. The fact that the cancer has arisen by chance can also not be excluded, but based on the extremely young age of the patient we consider this very unlikely.

No additional germline mutations in *MYD88* were found in our cohort of 126 patients suspected of hereditary gastric cancer. This implies that germline mutations in *MYD88* in GC patients are rare. Rather than screening complete cohorts of patients for this type of mutations, we suggest more thorough anamnestic analysis of GC patients to unravel whether a (partial) immunodeficiency could be causal for GC development.

In conclusion, we identified a functionally relevant homozygous missense variant in *MYD88* and a defective Th17 response in a patient with recurrent infections and early-onset gastric cancer. The missense variant in *MYD88* is the likely cause of persistent fungal infections that ultimately may have led to the development of early-onset gastric cancer in this patient. Future research is needed to unravel whether such immune deficiencies are a more common genetic risk factor for gastric cancer development.

## Appendix

International Gastric Cancer Genetics Group: Jan Lubinski, Department of Genetics and Pathology, Pomeranian Medical University, Szczecin, Poland; Anna Jakubowska, Department of Genetics and Pathology, Pomeranian Medical University, Szczecin, Poland; Urszula Teodorczyk, Department of Genetics and Pathology, Pomeranian Medical University, Szczecin, Poland; Hans K. Schackert, Department of Surgical Research, Universitätsklinikum Carl Gustav Carus, Technische Universität Dresden, Dresden, Germany; Cora M. Aalfs, Department of Clinical Genetics, Academic Medical Centre, Amsterdam, The Netherlands; Encarna B. Gómez García, Department of Clinical Genetics, Maastricht University Medical Center, Maastricht, The Netherlands; Guglielmina N. Ranzani, Department of Biology and Biotechnology, University of Pavia, Pavia, Italy; Valeria Molinaro, Department of Biology and Biotechnology, University of Pavia, Pavia, Italy; Liselotte P. van Hest, Department of Clinical Genetics, VU University Medical Center, Amsterdam, The Netherlands; Frederik J. Hes, Department of Clinical Genetics, Leiden University Medical Center, Leiden, The Netherlands; Elke Holinski-Feder, Medical Genetics Center, München, Germany; Maurizio Genuardi, Institute of Medical Genetics, Catholic University, Rome, Italy; Margreet G.E.M. Ausems, Department of Medical Genetics, University Medical Center Utrecht, The Netherlands; Rolf H. Sijmons, Department of Genetics, University Medical Center Groningen, University of Groningen, The Netherlands; Anja Wagner, Department of Clinical Genetics, Erasmus University Medical Center, Rotterdam, The Netherlands; Lizet E. van der Kolk, Family Cancer Clinic, The Netherlands Cancer Institute, Amsterdam, The Netherlands; Hugo Pinheiro, Expression Regulation in Cancer Group, IPATIMUP, Institute of Molecular Pathology and Immunology of the University of Porto, Porto, Portugal; Carla Oliveira, Expression Regulation in Cancer Group, IPATIMUP, Institute of Molecular Pathology and Immunology of the University of Porto/Department of Pathology and Oncology, Medical Faculty of the University of Porto, Porto, Portugal; Inga Bjørnevoll, Department of Medical Genetics and Pathology, St. Olavs University Hospital, Trondheim, Norway; Hildegunn Høberg Vetti, Western Norway Familial Cancer Center, Center for Medical Genetics and Molecular Medicine, Haukeland University Hospital, Bergen, Norway; J. Han J.M. van Krieken, Department of Pathology, Radboud university medical center, Nijmegen, The Netherlands.
